# Selenoprotein S and the Causal Risk of Hypertension in Pregnancy: A Mendelian Randomization Study

**DOI:** 10.3390/healthcare13182383

**Published:** 2025-09-22

**Authors:** Mengqi Cai, Wenrui Lv, Yan He, Weili Liu, Yuzhen Gao

**Affiliations:** 1 Jiangsu Key Laboratory of Preventive and Translational Medicine for Geriatric Diseases, Department of Epidemiology, School of Public Health, Suzhou Medical College, Soochow University, Suzhou 215123, China; mengqicai0920@163.com (M.C.); yhe@suda.edu.cn (Y.H.); 2 Department of Forensic Medicine, Medical College, Soochow University, Suzhou 215123, China; lvwenrui@suda.edu.cn

**Keywords:** selenoprotein S, selenium, pregnancy induced hypertension, Mendelian randomization

## Abstract

**Background:** Pregnancy-induced hypertension (PIH) affects approximately 10% of pregnancies worldwide, representing a leading cause of maternal and perinatal morbidity and mortality. The relationship between plasma selenium levels and PIH remains controversial, with observational studies limited by confounding factors. Selenoprotein S (SELENOS) has emerged as a potential biomarker for PIH risk. As one of the carrier proteins for dietary selenium, SELENOS plays a crucial role in oxidative stress and inflammatory pathways. However, the causal relationship between the plasma levels of the SELENOS and PIH development remains unclear. This study employed Mendelian randomization (MR) to investigate the causal link between the plasma levels of the SELENOS and PIH risk, providing evidence for preventive strategies. **Methods:** We conducted a two-sample MR analysis using genome-wide association study (GWAS) summary statistics from the INTERVAL study and FinnGen consortium. The analysis included individuals of European ancestry, utilizing the inverse-variance weighted (IVW) method as the primary approach. Comprehensive sensitivity analyses were performed to address potential pleiotropy and strengthen causal inference. **Results:** The analysis encompassed 3301 samples for the plasma levels of the SELENOS and 7686 PIH cases, 1109 pre-existing hypertension (PEH) cases, 4255 gestational hypertension (GH) cases, and 83 preeclampsia (PE) cases superimposed on chronic hypertension, alongside approximately 115,000 controls. Genetic variabilities that have been found to be accompanied by elevated levels of plasma selenioprotein levels showed significant associations with increased risk of PIH [odds ratio (OR) 1.078, 95% confidence interval (CI) 1.031–1.126, *p* = 0.001], PEH (OR 1.232, 95% CI 1.105–1.373, *p* < 0.001), and GH (OR 1.111, 95% CI 1.047–1.180, *p* = 0.001), with suggestive associations for preeclampsia superimposed on chronic hypertension (OR 1.590, 95% CI 1.078–2.344, *p* = 0.019). **Conclusions:** This study provides robust genetic evidence for a causal relationship between the plasma levels of the SELENOS and PIH risk, establishing SELENOS as a potential modifiable risk factor with significant clinical implications. These findings support the development of personalized selenium management strategies during pregnancy and highlight the potential for early screening and targeted interventions to improve maternal and fetal outcomes.

## 1. Introduction

Pregnancy-induced hypertension (PIH), encompassing various hypertensive disorders related to gestation, is the most common pregnancy complication, affecting approximately 10% of pregnancies worldwide. It also represents a major cause of maternal, fetal, and neonatal morbidity and mortality [1]. PIH includes subtypes such as hypertension developing before pregnancy or during pregnancy, and preeclampsia (PE) [2,3]. Gestational hypertension (GH) is characterized by elevated blood pressure (BP) (>140/90 mmHg) occurring after 20 weeks of gestation in previously normotensive women [4]. PE is defined as hypertension accompanied by significant proteinuria after 20 weeks of gestation [4]. Chronic hypertension, or pre-existing hypertension (PEH), refers to hypertension existing before pregnancy or before 20 weeks of gestation, impacting 1–2% of pregnancies. It represents the primary risk factor for developing PE, with approximately 20% of affected women progressing to superimposed PE [5]. PIHs are associated with sleep-disordered breathing [6], severe proteinuria, disseminated intravascular coagulation (DIC), HELLP syndrome, and an increased risk of maternal death [7,8]; fetal complications include intrauterine growth restriction and intrauterine fetal death, while neonatal complications include preterm birth, death, and low birth weight [9]. Additionally, PIHs serves as a lifelong cardiovascular risk marker. Patients with PIHs have an increased risk of acute heart failure, myocardial infarction, and ischemic stroke in the long term [10], as well as the elevated risk of stroke, chronic kidney disease, and multimorbidity [11], particularly among those with PE [2]. Therefore, the identification and evaluation of causal risk factors are crucial for the prevention and management of PIHs. Given its staged progression, early interventions may improve clinical outcomes.

Selenium in the human body is predominantly obtained through dietary sources rich in this essential trace element, with the selenium content in foodstuffs fundamentally determined by its bioavailability in soil [12]. Selenium exerts critical physiological functions in the human body primarily by modulating redox homeostasis, endoplasmic reticulum (ER) stability and inflammatory pathways [13], with its effects exhibiting a characteristic dose-dependent threshold. When the whole blood selenium concentration reaches 100 μg/L to 120 μg/L, corresponding to selenoprotein P (SELENOP) saturation, it effectively supports the aforementioned physiological functions [14]. The World Health Organization (WHO) recommends a daily selenium intake of 55 μg/d for adults [13], with insufficient intake potentially leading to weakened immune function, thyroid and reproductive dysfunction, and increased susceptibility to cardiovascular diseases [15]. Conversely, excessive selenium intake beyond physiological requirements may precipitate toxicological manifestations, including hematological abnormalities, alopecia, onycholysis, and neurological disturbances [16]. Notably, epidemiological studies have identified a positive correlation between selenium overconsumption and increased diabetes risk specifically in Caucasian populations [17]. Collectively, maintaining selenium intake within this narrow safety window proves critical for disease prevention, as deviations in either direction could trigger pathological risks. The pleiotropic biological activities of selenium are primarily mediated through selenocysteine-containing selenoproteins [18,19], with selenoprotein S (SELENOS) representing one functionally significant member of this protein family.

Empirical evidence from multiple observational studies suggests potential associations between aberrant SELENOS levels and PIHs [20,21]. A cross-sectional prospective study involving 32 pregnant women with PE revealed significantly lower serum selenium levels in these patients compared to healthy pregnant women and non-pregnant women [22]. A retrospective study conducted on a large Norwegian case–control cohort identified SELENOS g.-105G as a significant risk factor for PE in this population [23]. However, the association between SELENOS and PIHs is susceptible to confounding by factors such as dietary habits, supplement use, and socioeconomic status during pregnancy, which observational studies cannot eliminate, sometimes leading to contradictory conclusions. A study utilized data from the Norwegian Mother, Father, and Child Cohort Study (MoBa), which included 69,972 singleton pregnancies and conducted a questionnaire-based assessment. The results showed no significant association between selenium supplementation, serum selenium status, and PIH or its subtypes [24]. On this issue, Mendelian randomization (MR) analysis thus offers a novel method to evaluate the causal relationship between plasma SELENOS levels and PIHs.

In MR, genetic variants act as instrumental variables (IVs) for exposures to infer causality between exposures and outcomes, enabling the investigation of potential causal relationships while mitigating confounding factors. Bidirectional MR tests causal directions between two traits using separate genetic instruments for each trait, resolving ambiguity in observational associations [25,26]. This study utilized a two-sample bidirectional MR approach with Genome-wide association study (GWAS) summary statistics of SELENOS from the INTERVAL study and summary statistics of PIH and its subtypes from the FinnGen consortium to ascertain causality between genetically predicted plasma SELENOS levels and PIHs. Subsequently, comprehensive assessments for heterogeneity, pleiotropy, and sensitivity were conducted to evaluate the robustness of the analysis.

## 2. Materials and Methods

### 2.1. Study Design

**Figure 1:**
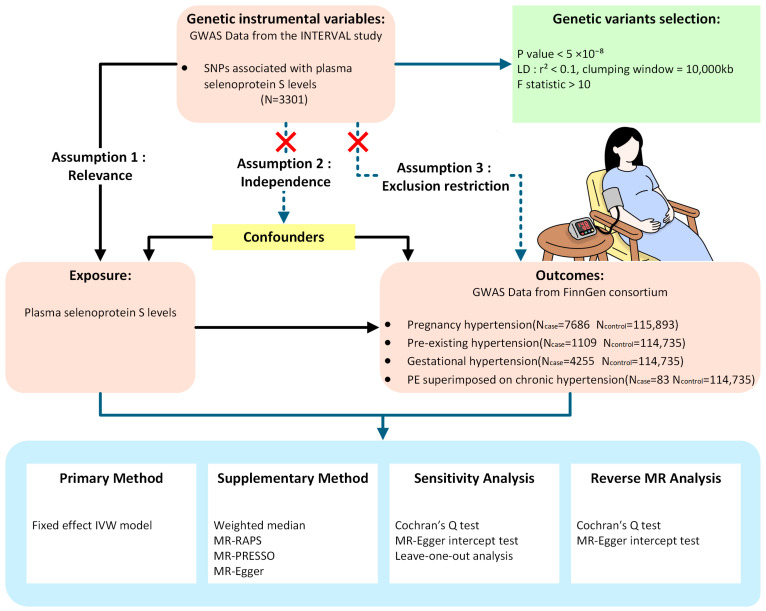
Study design for the Mendelian randomization analysis of plasma SELENOS levels and the risks of PIHs.

This workflow of the study was illustrated in Figure 1. This study utilized publicly available, de-identified summary statistics from existing GWAS. This two-sample MR investigation focused on the relationship between plasma SELENOS levels and PIHs. Outcomes included PIH, PEH, GH and PE superimposed on chronic hypertension. The validity of MR analysis is predicated upon satisfying three fundamental assumptions. Firstly, a robust association must exist between the genetic variants and the exposure factor (relevance assumption). Secondly, the selected genetic instruments must demonstrate independence from confounding factors (independence assumption). Thirdly, these instruments must influence the outcome exclusively through their effect on the exposure, precluding alternative biological pathways (exclusion restriction assumption) [27,28,29]. The study identified the genetic tools indicating SELENOS levels and evaluated their statistical power using *F*-statistics. Multiple sensitivity analyses, including weighted median, MR Robust Adjusted Profile Scoring (MR-RAPS), MR Pleiotropy RESidual Sum and Outlier (MR-PRESSO) and MR-Egger, were conducted to verify the robustness of primary findings and evaluate the core MR assumptions [28,30].

### 2.2. Data Sources

GWAS summary statistics were sourced from the INTERVAL study and FinnGen consortium. These data are publicly accessible through the IEU database (https://gwas.mrcieu.ac.uk/datasets/ (accessed on 30 May 2025)). Only individuals of European ancestry were included to ensure robustness and minimize confounding from population stratification. Detailed data sources, sample sizes, and survey years are provided in Table S1. The data on SELENOS was obtained from Sun et al., who conducted genome-wide profiling of 2994 plasma proteins in a cohort of 3301 healthy individuals of European ancestry using data from the INTERVAL study. The INTERVAL study is a randomized controlled trial (RCT) jointly led by NHS Blood and Transplant and the Epidemiology Research Centre at the University of Oxford, involving over 50,000 blood donors from across the United Kingdom [31]. GWAS summary statistics of PIHs were obtained from the FinnGen consortium. The FinnGen consortium is a national large-scale genomics and population health research initiative launched in Finland, utilizing the advantageous characteristics of the Finnish population for genetic studies. The dataset consisted of 7686 cases and 115,893 controls for PIH, 1109 cases and 114,735 controls for PEH, 4255 cases and 114,735 controls for GH, 83 cases and 114,735 controls for PE superimposed on chronic hypertension.

### 2.3. Genetic Instruments

Genetic instrument selection was initiated by screening single-nucleotide polymorphisms (SNPs) achieving genome-wide significance (*p* < 5 × 10^−8^). Subsequent clumping procedures ensured independence among selected SNPs using a linkage disequilibrium (LD) threshold of r^2^ < 0.1 within 10,000 kb windows [32]. To harmonize exposure-outcome datasets, exclusion criteria comprised missing SNPs in GWAS summary statistics and palindromic SNPs with minor allele frequency exceeding 0.4. Moreover, we substituted missing SELENOS-associated SNPs in the database for PIH and its subtypes with proxy variants (r^2^ > 0.8) from the 1000 Genomes European reference panel. Instrument robustness was quantified via the *F*-statistic: *F* = (N − K − 1) × R^2^/K × R^2^ (1 − R^2^), where R^2^ denotes phenotypic variance explained by genetic variants, N reflects exposure GWAS sample size, and K indicates instrument count [33]. *F*-statistics exceeding 10 mitigated weak instrument bias concerns in subsequent MR analyses.

### 2.4. Statistical Analysis

The primary analytical approach employed inverse-variance weighted (IVW) analysis with Bonferroni correction for multiple testing [34]. Under the IVW framework’s core assumption of IVs exhibiting null pleiotropic effects, we employed Cochran’s Q statistic to quantify heterogeneity across genetic instruments. This approach ensures that any potential confounding factors and biases are appropriately addressed, enabling robust conclusions regarding the causal relationship under investigation. To strengthen causal inference robustness and address potential violations of MR assumptions, comprehensive sensitivity analyses were conducted including weighted median, MR-RAPS, MR-PRESSO, MR-Egger regression, and leave-one-out validation [30,35,36].

Weighted median estimator derives causal effects from the median of precision ordered IV estimates, providing consistent estimates when ≥50% of IVs are valid. MR-RAPS enhances result reliability in high-heterogeneity settings by modeling and adjusting for pleiotropic effects. MR-PRESSO specifically detects and corrects horizontal pleiotropy through outlier identification followed by effect size recalculation. MR-Egger regression assesses directional horizontal pleiotropy via its intercept term, where a non-zero intercept indicates systematic pleiotropy, warranting cautious interpretation of IVW results. Leave-one-out analysis evaluates individual genetic variant influences on overall effect estimates by systematically excluding single SNPs. Through comprehensive application of these methods, potential biases from outliers, heterogeneity, and directional pleiotropy can be systematically evaluated, thereby enhancing the robustness and reliability of findings.

Results are presented as odds ratios (ORs) with 95% confidence intervals (95% CIs). For all outcomes, a Bonferroni-corrected significance level of 2-sided *p* < 0.013 (0.05/4 [4 types of PIHs]) was considered statistically significant. Statistical power was determined using the mRnd online platform (https://shiny.cnsgenomics.com/mRnd/, accessed on 30 May 2025). All analyses were implemented in R programming environment, specifically version 4.2.1, as provided by the R Development Core Team (Vienna, Austria), utilizing the ‘TwoSampleMR’ and ‘MRPRESSO’ packages.

## 3. Results

### 3.1. Genetic Instruments and Statistical Power Assessment

The genetic instruments selected for this MR analysis, comprising 15 distinct SNPs on chromosome 19 were designated as proxies for SELENOS (Table 1). All SNPs demonstrated robust instrument strength, with *F*-statistics substantially exceeding the weak instrument threshold of 10 and average *F* = 89.99, effectively mitigating weak instrument bias concerns. Collectively, these SNPs explained 37.89% of the variance in plasma SELENOS levels, indicating significant phenotypic influence. Power analysis for the MR framework confirmed adequate statistical power to detect causal effects between SELENOS and PIHs outcomes, supporting the analytical validity of the IVs assumptions and the integrity of causal inference pathways (Table S2).

### 3.2. Primary MR Findings

**Figure 2:**
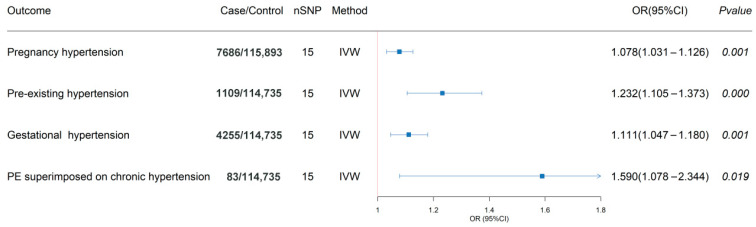
Forest plots for the associations of plasma selenoprotein S levels with risks of pregnancy-associated hypertension in the main inverse-variance weighted Mendelian randomization. Solid squares represent the point estimates of effect sizes (odds ratios) for each SNP on disease risk. Horizontal lines indicate the corresponding 95% confidence intervals for these effect estimates. An observed 2-sided *p* < 0.013 after Bonferroni correction (0.05/4 [1 exposure and 4 outcomes]) was considered to be statistically significant, whereas a 2-sided *p* value between 0.013 and 0.05 was considered suggestively significant; OR, odds ratio; SNP, single-nucleotide polymorphism.

**Figure 3:**
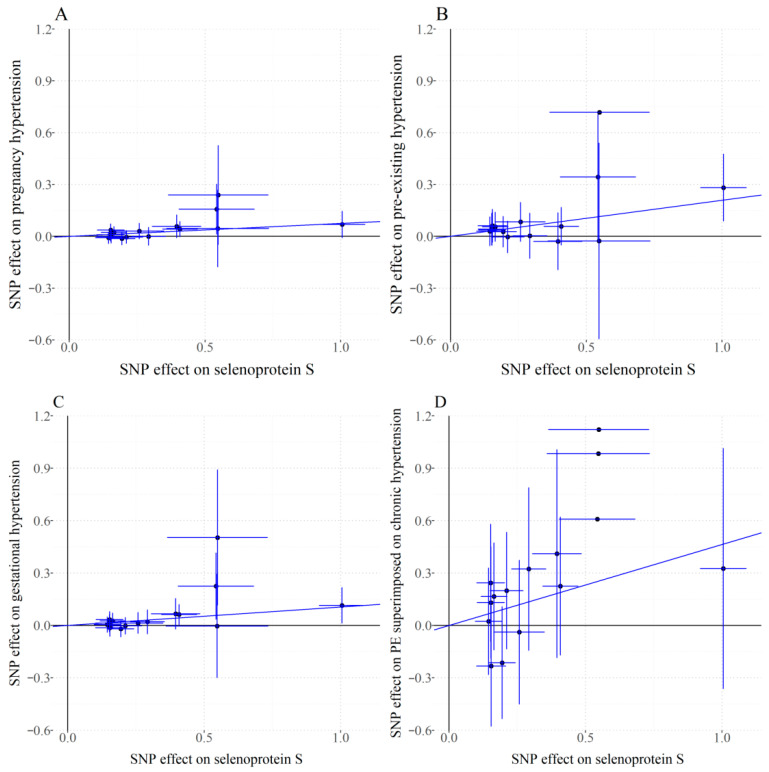
Scatterplots for associations of the plasma levels of selenoprotein S with risks of pregnancy-associated hypertension. The slope of the line indicates the estimate of effect using the inverse-variance weighted method. Circles indicate marginal genetic associations with selenoprotein S level and risk of outcome for each variant. Horizontal and vertical lines through each circle represent the corresponding 95% CIs for the genetic associations. *X*-axis: SNP effect on selenoprotein S plasma level (β per allele). *Y*-axis: SNP effect on pregnancy-associated hypertension (log OR per allele). (**A**) Pregnancy hypertension. (**B**) Pre-existing hypertension. (**C**) Gestational hypertension. (**D**) PE superimposed on chronic hypertension. PE superimposed on chronic hypertension indicates pre-eclampsia superimposed on chronic hypertension; SNP indicates single-nucleotide polymorphism.

According to the Cochran Q test results, for the analysis of PIH, PEH, GH and PE superimposed on chronic hypertension, no significant heterogeneity was found among the genetic tools (*p* > 0.05), supporting the use of fixed-effect models. MR-Egger intercept tests indicated no evidence of horizontal pleiotropy (*p* > 0.05), confirming that instrument variables influenced PIHs risk exclusively through SELENOS pathways, thereby satisfying core MR assumptions (Table S3). IVW analysis demonstrated statistically significant associations between genetically predicted plasma SELENOS levels and increased risk of PIH (OR 1.078, 95% CI 1.031–1.126, *p* = 0.001), PEH (OR 1.232, 95% CI 1.105–1.373, *p* < 0.001), and GH (OR 1.111, 95% CI 1.047–1.180, *p* = 0.001), along with suggestive significance for PE superimposed on chronic hypertension (OR 1.590, 95% CI 1.078–2.344, *p* = 0.019), while the association did not reach the Bonferroni-corrected significance levels (Figure 2). Figure 3 delineates the relationship between individual genetic variants, plasma SELENOS levels, and hypertension risks.

### 3.3. Sensitivity Analysis

Four complementary sensitivity analyses-weighted median, MR-RAPS, MR-PRESSO, and MR-Egger methods-convergently confirmed the significant association between plasma SELENOS levels and PIHs observed in primary analyses (Table 2). Consistent effect directions emerged across PIH, PEH, and GH outcomes, with all *p*-values below the Bonferroni corrected significance threshold except for suggestive associations in PIH by weighted median and PEH by MR-Egger, robustly substantiating that altered SELENOS levels elevate gestational hypertension risk. Regarding PE superimposed on chronic hypertension, only MR-RAPS (*p* = 0.020) and MR-PRESSO (*p* = 0.022) showed suggestive significance, whereas weighted median (*p* = 0.075) and MR-Egger (*p* = 0.115) exceeded statistical significance thresholds. Leave-one-out analysis confirmed that all SNPs had no explicit influence on the observed associations (Figures S1–S4). Steiger directionality testing excluded reverse causality for all the outcomes relative to SELENOS (*P*_Steiger_ < 0.05, Table S4). Finally, reverse MR analyses using IVW universally yielded non-significant effects, thus validating the causal direction from SELENOS to PIHs (Table S5). Sensitivity analyses provided robust validation for the primary findings, demonstrating that genetically predicted SELENOS levels was associated with increased risks of PIH, GH, PEH and PE superimposed on chronic hypertension. These associations remained consistent across all sensitivity analyses, which confirmed the absence of significant pleiotropy or heterogeneity and suggested that no single IV disproportionately influenced the outcomes.

## 4. Discussion

### 4.1. Primary Findings

This two-sample MR study systematically evaluated the relationship between genetically predicted plasma SELENOS levels and the risk of PIHs, including key subtypes. Our findings support a potential causal association between plasma SELENOS levels and the risk of PIHs, and the results remained robust across multiple sensitivity analyses that addressed different assumptions of horizontal pleiotropy, which align with prior observational reports, linking dysregulated SELENOS expression with PIH and its subtypes.

### 4.2. Our Findings in the Context of Current Literature

Multiple complex pathophysiological mechanisms have been proposed to elucidate the development of PIHs. The pathogenesis initiates with abnormal placentation, where impaired trophoblast invasion leads to failure of uterine spiral artery remodeling, resulting in narrowed high-resistance vessels, insufficient placental perfusion, and localized ischemia-hypoxia [37]. Placental ischemia subsequently triggers the release of pro-inflammatory factors that damage the maternal circulatory system [38]. Excessive production of reactive oxygen species (ROS) secondary to reduced placental perfusion causes oxidative stress [39]. PIHs also induces aberrant activation of AT1-mediated signaling, enhancing the angiotensin II cascade [38]. PE, a severe subtype, is strongly associated with placenta-derived soluble fms-like tyrosine kinase-1 (sFlt-1). Superfluous sFlt-1 binds vascular endothelial growth factor (VEGF), preventing VEGF interaction with endothelial cell surface receptors, thereby causing endothelial dysfunction [40].

Functioning as a significant effector in selenium metabolic pathways, SELENOS levels are modulated by genetic and environmental determinants, critically regulating vascular homeostasis and oxidative stress responses during gestation [41]. SELENOS is an ER transmembrane protein that serves as a core component of the ER-associated degradation (ERAD) pathway. During ER stress regulation, SELENOS mediates the translocation of misfolded proteins from the ER to the cytoplasm by interacting with the p97 complex, ultimately leading to their proteasomal degradation [42]. When selenium deficiency downregulates SELENOS expression, misfolded proteins accumulate within the ER and triggered the unfolded protein response (UPR) to rigorously maintain protein fidelity [41]. Additionally, SELENOS protects cells against oxidative damage and modulates ER stress-induced apoptosis [18]. The pathophysiological core of PIHs involves a cascade reaction initiated by insufficient placental perfusion. Reduced plasma SELENOS levels activate the UPR, exacerbate endothelial damage and diminish anti-inflammatory capacity simultaneously to promote both abnormal placental formation in early pregnancy and oxidative stress with endothelial injury during mid-late gestation. This inflammatory cascade further leads to multi-organ involvement.

However, our study indicates that elevated SELENOS levels also contribute to an increased risk of PIHs. Given the narrow safe intake window of SELENOS, this suggests that the dose-dependent U-shaped effect of SELENOS on the pathogenesis and progression of PIHs is not merely protective. IVW results revealed that the effect of genetically predicted elevated SELENOS levels on PEH (OR = 1.232) was stronger than that on GH (OR = 1.111), with the most pronounced effect observed in cases with combined PE (OR 1.590). A plausible explanation is that within a moderate range, SELENOS exerts protective effects by regulating oxidative stress, endoplasmic reticulum homeostasis, and inflammatory pathways. Conversely, when levels exceed a critical threshold, it may exacerbate PIHs risk by overactivating pathological pathways. Additionally, since GH is less severe pathologically compared to PE, the impact of elevated SELENOS levels is more pronounced in PE. However, we must acknowledge the limitation of insufficient statistical power due to the small number of cases involving PE superimposed on chronic hypertension.

Further mechanistic studies have shown that in pregnant women with low selenium levels, increasing selenoprotein concentrations through selenium supplementation can reduce the risk of PIHs [12]. In a RCT involving 60 high-risk pregnant women, selenium supplementation significantly increased the total antioxidant capacity compares to a placebo (β = 82.88 mmol/L, 95% CI 3.03–162.73, *p* = 0.04) [20]. A RCT involving 230 pregnant women in the UK demonstrated that pregnant women with low selenium status have lower PE risk if they have selenium supplementation [43]. Concurrent evidence indicates selenium’s beneficial impact on embryonic development and viability, whereas deficiency correlates with adverse pregnancy outcomes [44]. Neonatal birth weight exhibits significant positive correlation with maternal plasma selenium (r = 0.47, *p* < 0.001) [45]. A Dutch cross-sectional study of 1129 pregnant women demonstrated significantly lower serum selenium levels at 12 weeks’ gestation among preterm deliveries compared to term deliveries [46].

Despite these promising associations, no formal clinical implementation guidelines exist for selenium supplementation during pregnancy [47]. Aspirin remains the sole recommended pharmacological prophylaxis for PE prevention [48,49]. Based on the dynamics of selenium metabolism during pregnancy, screening for serum selenium thresholds in the first trimester is recommended as an indicator for supplementation. Selenium supplementation promotes the restoration of SELENOS activity and enhances its anti-inflammatory effects. A SPRINT study involving 227 pregnant women in the UK revealed that women carrying the minor A allele of rs3877899 demonstrated enhanced capacity to maintain selenium levels during gestation, consequently requiring a lower threshold for additional selenium supplementation [50].Therefore, similarly, when developing dynamic monitoring models for pregnancy, it is recommended to set higher selenium supplementation thresholds for pregnant women carrying alleles that reduce the SELENOS levels, while requiring lower upper limits for selenium intake in those with alleles associated with increased SELENOS levels. In regions with low selenium levels, increasing the consumption of selenium-rich foods such as fish and shellfish is advised [51]. However, since excessive selenium can also cause potential harmful effects [52], high-dose selenium-containing foods like Brazil nuts warrant caution [53].

### 4.3. Strengths and Limitations

The key strength of this study lies in its application of MR, leveraging the inherent randomization of genetic instruments to address confounding and reverse causality inherent in observational studies. The findings provide compelling evidence for the causal relationship between plasma SELENOS levels and the risk of PIHs, with the validity of results reinforced through robust statistical analyses. However, interpreting results requires careful consideration of limitations. Firstly, all 15 instrumental variables are located within a gene-dense region on chromosome 19, encompassing not only SELENOS but also BCAM, BCL3, APOE, APOC1, APOC4, and NECTIN2. While our statistical tests (MR-Egger intercept and MR-PRESSO) did not detect significant horizontal pleiotropy, the biological plausibility of pleiotropy through nearby genes cannot be fully excluded. This is particularly relevant given APOE’s established roles in lipid metabolism and vascular function, which could independently influence hypertension risk. The genomic clustering of our instrumental variables necessitates cautious interpretation of our findings and emphasizes the critical need for replication using independent genetic instruments, ideally from trans-acting variants that regulate SELENOS expression through different biological pathways. Secondly, caution is needed when interpreting the statistical power for mediation effects across different types of PIHs, especially in PE superimposed on chronic hypertension. The large standard error of the total effect suggests limited precision, likely due to sample size constraints. Thirdly, genetic instruments for SELENOS status reflect average levels during blood collection periods rather than trimester-specific exposures, limiting the assessment of SELENOS dynamics during distinct gestational stages. Lastly, the reliance on GWAS data primarily from European-ancestry populations hinders generalizability to other ancestral groups. To enhance the generalizability of the research, studies involving more diverse population-based cohorts are required.

### 4.4. Further Implications

Future research should prioritize the following aspects: validating these associations through multi-ethnic population studies while expanding the selection criteria for cases. These findings require verification in larger cohorts to confirm their clinical relevance, particularly for the PE superimposed on chronic hypertension subtype. Future studies should prioritize the identification of additional, geographically dispersed genetic variants that influence SELENOS levels to strengthen causal inference. Furthermore, developing SELENOS-level-targeted therapeutic strategies. Finally, elucidating the pathophysiological mechanisms of PIHs. Implementing this roadmap will bridge experimental evidence and clinical management, reducing PIHs-related morbidity.

## 5. Conclusions

The results of this study using two-sample MR reveal a potential causal relationship between SELENOS and the risk of PIHs. These findings highlight SELENOS as a modifiable risk factor for the development of PIHs, warranting further investigation into its pathological mechanisms. In line with previous observational data linking disrupted selenium metabolism to gestational hypertension disorders, our study emphasizes the clinical significance of monitoring maternal SELENOS levels. Early detection of abnormal SELENOS levels, along with regulation of selenium intake, may concomitantly enhance selenium metabolic equilibrium and pregnancy-related blood pressure outcomes. Future RCTs should meticulously assess the effects of regulation of selenium intake on PIHs incidence and the overall health of mothers and infants, offering conclusive evidence to advance personalized preventive strategies.

## Figures and Tables

**Table 1:** Characteristics of 15 genetic variants used as instrumental variables for plasma selenoprotein S.

SNP	Chr	Position (Build 37)	Nearest Gene	EA	OA	β	SE	*p* Value	F Statistic
rs117261169	19	45491032	CLPTM1	T	C	−0.55	0.1	1.20 × 10^8^	37.43
rs2965169	19	45251156	BCL3	C	A	−0.15	0.03	2.04 × 10^9^	37.06
rs151330717	19	45196964	CEACAM16	A	G	−0.55	0.09	5.13 × 10^9^	39.63
rs405509	19	45408836	APOE	G	T	−0.17	0.02	1.45 × 10^11^	45.36
rs28399657	19	45318351	BCAM	G	A	−0.54	0.07	1.78 × 10^14^	60.4
rs28399637	19	45324138	BCAM	A	G	0.15	0.03	5.62 × 10^9^	33.87
rs11668327	19	45398633	TOMM40	C	G	−0.29	0.03	8.32 × 10^20^	84.7
rs429358	19	45411941	APOE	C	T	0.41	0.03	5.50 × 10^35^	148.84
rs6859	19	45382034	NECTIN2	G	A	−0.15	0.03	5.01 × 10^9^	34.1
rs7412	19	45412079	APOE	T	C	−1.01	0.04	1.78 × 10^120^	559.13
rs4803759	19	45327459	BCAM	C	T	0.15	0.03	1.35 × 10^8^	33.26
rs62117161	19	45233385	BCL3	G	A	−0.4	0.05	5.62 × 10^18^	77.13
rs60049679	19	45429708	APOC1	C	G	0.26	0.05	3.31 × 10^8^	32.35
rs4263041	19	45438643	APOC4	G	A	−0.21	0.03	6.92 × 10^12^	64.1
rs7343130	19	45331103	BCAM	G	A	0.19	0.03	5.25 × 10^15^	62.52

SNP, single-nucleotide polymorphism; Chr, chromosome; EA, effect allele; OA, other allele.

**Table 2:** Sensitivity analyses for associations of plasma selenoprotein S levels with pregnancy-associated hypertension and blood pressure.

Outcome	NO. SNPs	Weighted Median		MR-RAPS		MR-PRESSO		MR-Egger	
		OR (95% CI)	*p* Value	OR (95% CI)	*p* Value	OR (95% CI)	*p* Value	OR (95% CI)	*p* Value
**Pregnancy hypertension**	15	1.076 (1.014, 1.143)	0.016	1.078 (1.031, 1.128)	9.18 × 10^4^	1.078 (1.024, 1.134)	0.004	1.109 (1.023, 1.202)	0.012
Pre-existing hypertension	15	1.301 (1.127, 1.501)	3.28 × 10^4^	1.234 (1.104, 1.379)	2.18 × 10^4^	1.232 (1.096, 1.384)	4.70 × 10^−4^	1.265 (1.035, 1.546)	0.022
Gestational hypertension	15	1.120 (1.036, 1.210)	0.004	1.112 (1.049, 1.180)	3.87 × 10^4^	1.111 (1.034, 1.193)	0.004	1.182 (1.062, 1.315)	0.002
PE superimposed on chronic hypertension	15	1.606 (0.953, 2.707)	0.075	1.598 (1.076, 2.372)	0.02	1.590 (1.069, 2.363)	0.022	1.773 (0.869, 3.615)	0.115

*p* < 0.013 after Bonferroni correction (0.05/4 [1 exposure and 4 outcomes]) was considered to be statistically significant.

## Data Availability

The exposure data used in this study can be retrieved and accessed from the website https://gwas.mrcieu.ac.uk/datasets/ (accessed on 30 May 2025), and the outcome data can be retrieved and accessed from the website https://r12.finngen.fi/ (accessed on 30 May 2025).

## Supplementary Materials

The following supporting information can be downloaded at: https://www.mdpi.com/article/10.3390/healthcare13182383/s1, Figure S1: Leave-one-out plot for the causal association between plasma selenoprotein S levels and pregnancy hypertension after omitting each SNP; Figure S2: Leave-one-out plot for the causal association between plasma selenoprotein S levels and pre-existing hypertension after omitting each SNP; Figure S3: Leave-one-out plot for the causal association between plasma selenoprotein S levels and gestational hypertension after omitting each SNP; Figure S4: Leave-one-out plot for the causal association between plasma selenoprotein S levels and pre-eclampsia superimposed on chronic hypertension after omitting each SNP; Table S1: Summary of the GWAS Data Used in the MR Analysis; Table S2: Description of the genetic instruments used in this Mendelian randomization study; Table S3: Pleiotropy and heterogeneity assessment for associations of plasma selenoprotein S levels with pregnancy-associated hypertension and inverse-variance-weighted model used in the analysis; Table S4: The results of the Mendelian randomization Steiger test; Table S5: Reverse IVW Mendelian randomization analyses for the associations of pregnancy-induced-hypertension with plasma selenoprotein S levels.

## Abbreviations

The following abbreviations are used in this manuscript:

SELENOS	selenoprotein S
PIH	pregnancy induced hypertension
MR	Mendelian randomization
GWAS	Genome-wide association study
IVW	inverse-variance weighted
PEH	pre-existing hypertension
GH	gestational hypertension
PE	Preeclampsia
OR	odds ratio
CI	confidence interval
BP	Blood pressure
DIC	disseminated intravascular coagulation
ER	endoplasmic reticulum
SELENOP	selenoprotein P
WHO	World Health Organization
MoBa	Norwegian Mother, Father and Child Cohort Study
IVs	instrumental variables
MR-RAPS	Mendelian Randomization Robust Adjusted Profile Scoring
MR-PRESSO	Mendelian Randomization Pleiotropy RESidual Sum and Outlier
RCT	randomized controlled trial
SNPs	Single-nucleotide polymorphisms
LD	linkage disequilibrium
ROS	reactive oxygen species
sFlt-1	soluble fms-like tyrosine kinase-1
VEGF	vascular endothelial growth factor
ERAD	ER-associated degradation
UPR	unfolded protein response
