# Supplementary material for: Selenoprotein S and the Causal Risk of Hypertension in Pregnancy: A Mendelian Randomization Study
Source: Healthcare (Basel). 2025 Sep 22;13(18):2383. doi: 10.3390/healthcare13182383 (PMC12469401; doi:10.3390/healthcare13182383)

Table S1. Summary of the GWAS Data Used in the MR Analysis.

Phenotype	No of participants (cases / controls)	Ancestry	Consortium/cohort	Author	Year of publication	PMID
SELENOS	3,301	European	The INTERVAL study	Sun et al	2018	29875488
Pregnancy hypertension	7,686 / 115,893	European	Finn Gen	-	2021	-
Pre-existing hypertension complicating pregnancy, childbirth and the puerperium	1,109 / 114,735	European	Finn Gen	-	2021	-
Gestational [pregnancy-induced] hypertension	4,255 / 114,735	European	Finn Gen	-	2021	-
Pre-eclampsia superimposed on chronic hypertension	83 / 114,735	European	Finn Gen	-	2021	-

SELENOS, Selenoprotein S.

Table S2. Description of the genetic instruments used in this Mendelian randomization study

Outcome	Number of SNPs	Variance* (%)	F-statistic†	Power at different ORs and α = 0.05			
				1.07	1.10	1.20	1.50
Pregnancy hypertension	15	37.89	89.99	0.95	1.00	1.00	1.00
Pre-existing hypertension	15	37.89	89.99	0.30	0.53	0.98	1.00
Gestational hypertension	15	37.89	89.99	0.79	0.98	1.00	1.00
PE superimposed on chronic hypertension	15	37.89	89.99	0.07	0.09	0.20	0.80

*Phenotypic variance explained by the genetic instruments used in the present Mendelian randomization analysis.

†Average F-statistic calculated with all included genetic instruments.

Table S3. Pleiotropy and heterogeneity assessment for associations of plasma selenoprotein S levels with pregnancy associated hypertension and inverse-variance-weighted model used in the analysis.

Outcomes	Cochran′s Q test		MR-Egger intercept		IVW Model
	Q value	*P*-value	β (se)	*P*-value	
Pregnancy hypertension	13.521	0.486	-0.009(0.011)	0.416	Fixed effect model
Pre-existing hypertension	9.635	0.788	-0.009(0.028)	0.762	Fixed effect model
Gestational hypertension	15.114	0.370	-0.020(0.015)	0.203	Fixed effect model
PE superimposed on chronic hypertension	11.533	0.644	-0.035(0.099)	0.726	Fixed effect model

An observed 2-sided P value <0.05 was considered nominal significance.

Table S4. The results of the Mendelian randomization Steiger test.

Outcome	R^2 for exposure	R^2 for outcome	Correct causal direction	P_{Steiger}
Pregnancy hypertension	0.379	2.00e-04	TRUE	0
Pre-existing hypertension	0.379	2.05e-04	TRUE	0
Gestational hypertension	0.379	2.35e-04	TRUE	0
PE superimposed on chronic hypertension	0.379	1.48e-04	TRUE	0

An observed 2-sided P<0.013 after Bonferroni correction (0.05/4 [one exposure and 4 outcomes]) was considered to be statistically significant.

Table S5. Reverse IVW Mendelian randomization analyses for the associations of pregnancy induuced hypertension with plasma selenoprotein S levels.

Exposure	NO. SNPs	β(95%CI)	*P*-value	Cochran′s Q test		MR-Egger intercept	
				Q value	*P*-value	β (se)	*P*-value
Pregnancy hypertension	40	0.014(-0.055, 0.083)	0.687	32.198	0.771	-0.001(0.009)	0.948
Pre-existing hypertension	16	-0.004 (-0.039, 0.032)	0.832	8.751	0.890	-0.001(0.011)	0.902
Gestational hypertension	34	0.018(-0.057, 0.093)	0.637	31.437	0.545	-0.009(0.015)	0.563
PE superimposed on chronic hypertension	18	0.002(-0.011, 0.015)	0.765	18.014	0.388	-0.002(0.022)	0.946

The SNPs that were identified to be significantly associated with exposures ($P < 1\times10^{-5}$ for Pregnancy hypertension, Pre-existing hypertension, Gestational hypertension and PE superimposed on chronic hypertension) and were not in linkage disequilibrium with other SNPs ($r^2 < 0.001$, with a 10,000 kb clumping window) were selected as genetic instruments for exposures. IVW, inverse-variance weighted; SNP, single-nucleotide polymorphism; 95% CI, 95% confidence interval.

Figure S1. Leave-one-out plot for the causal association between plasma selenoprotein S levels and pregnancy hypertension after omitting each SNP.

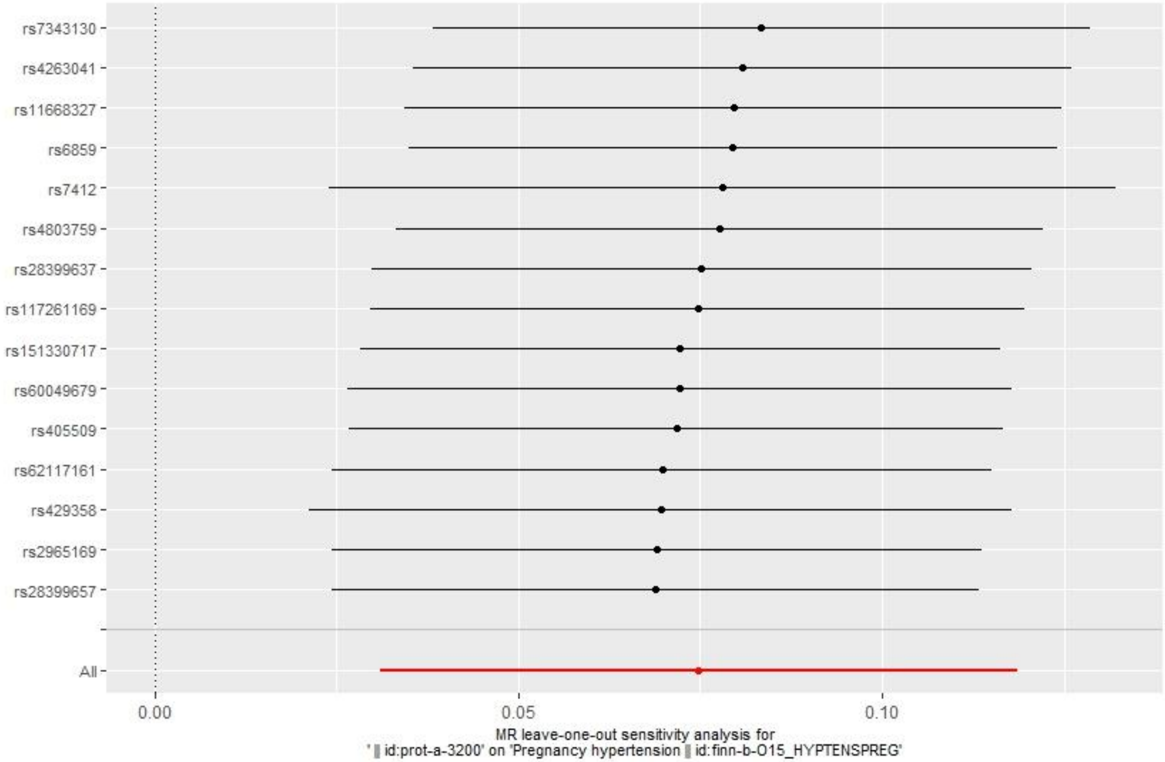

Figure S2. Leave-one-out plot for the causal association between plasma selenoprotein S levels and pre-existing hypertension after omitting each SNP.

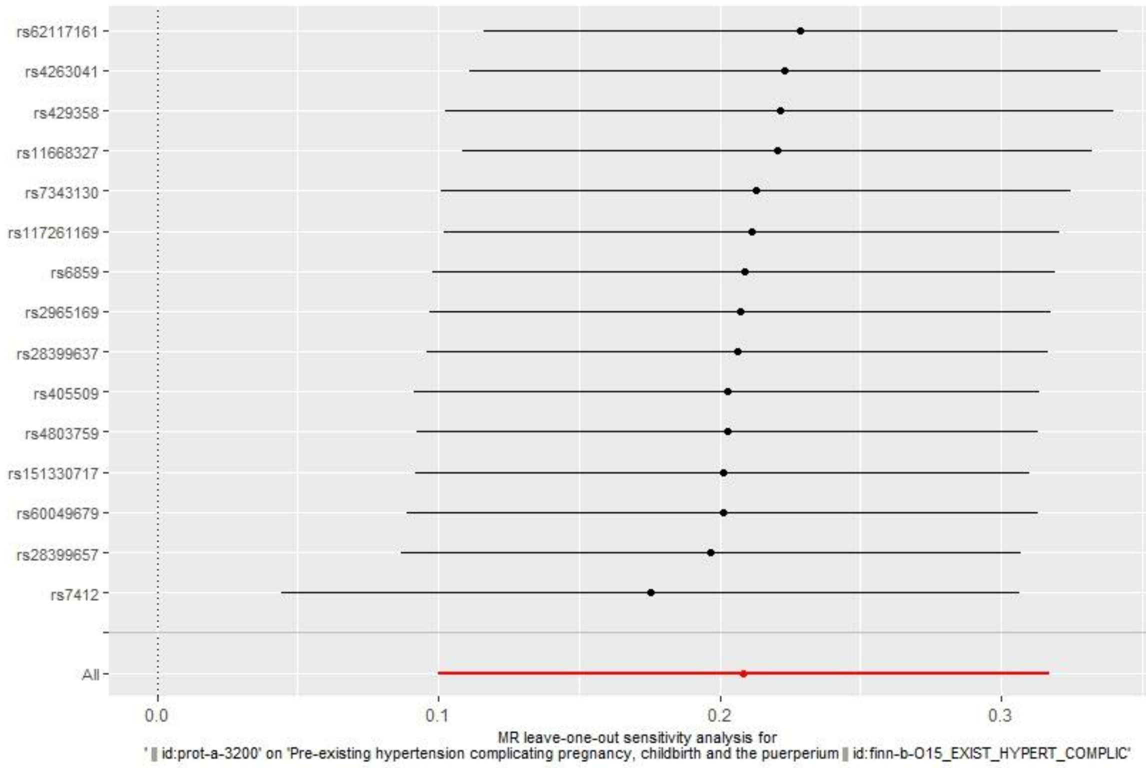

Figure S3. Leave-one-out plot for the causal association between plasma selenoprotein S levels and gestational hypertension after omitting each SNP.

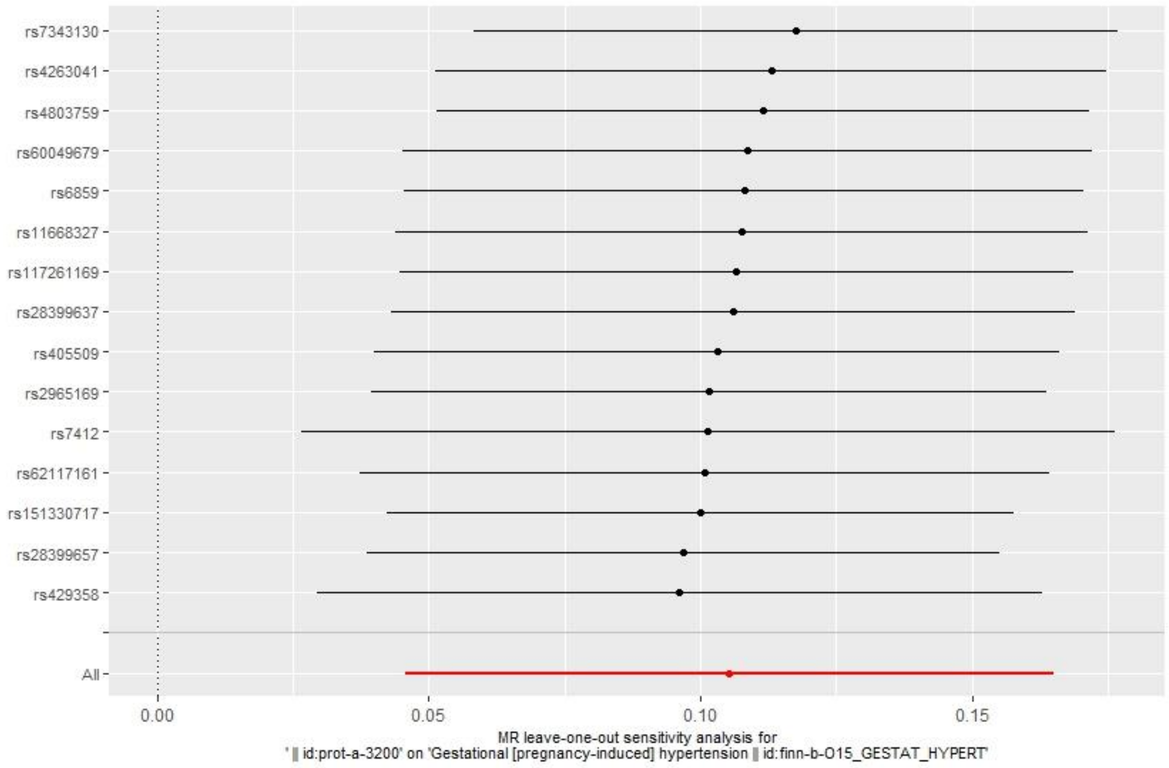

Figure S4. Leave-one-out plot for the causal association between plasma selenoprotein S levels and pre-eclampsia superimposed on chronic hypertension after omitting each SNP.

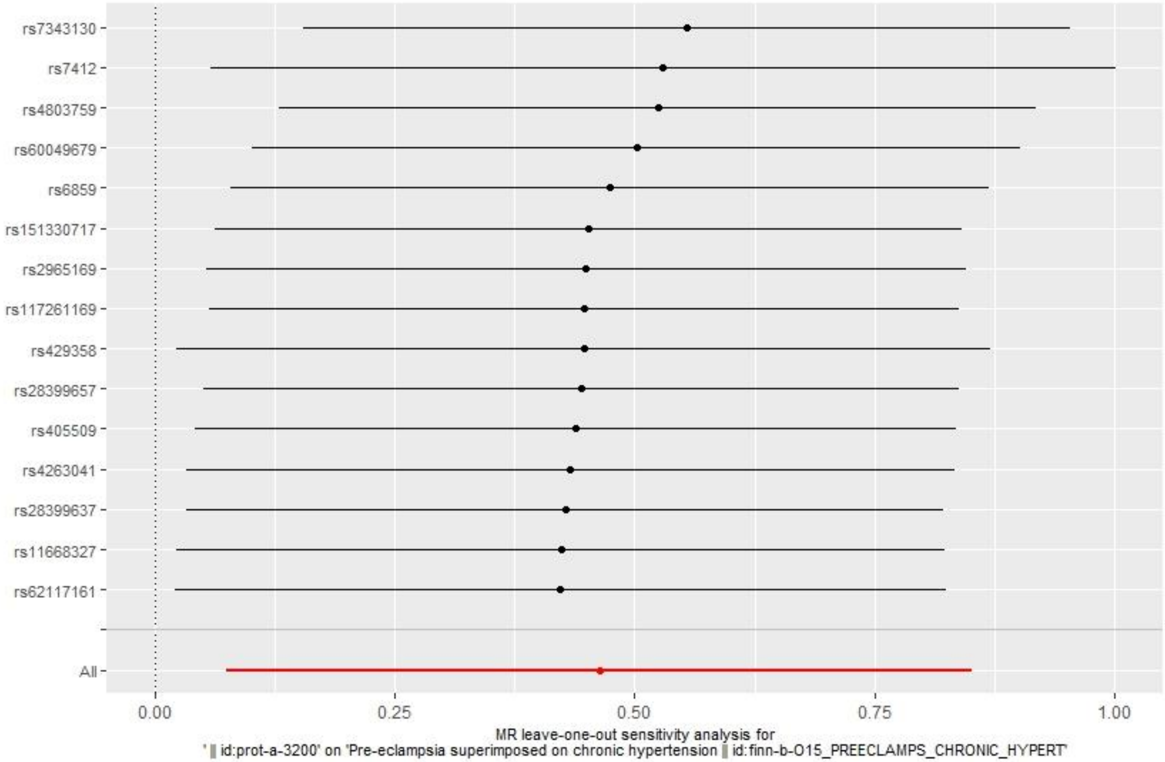

Supplement: Supplementary file 1 [file healthcare-13-02383-s001.zip › healthcare-3792529-supplementary.pdf]
